# Optimal Design and Analysis on High Overload Buffer Structure of Passive Semi-Strapdown Inertial Navigation System

**DOI:** 10.3390/s20041131

**Published:** 2020-02-19

**Authors:** Jinqiang Li, Jie Li, Li Qin, Wei Liu, Xiaokai Wei, Ning Gao, Yang Liu

**Affiliations:** 1 National Key Laboratory for Electronic Measurement Technology, North University of China, Taiyuan 030051, China; leejq0302@163.com (J.L.); weixiaokai1128@163.com (X.W.); gaoning_1995@163.com (N.G.); lylyly357@163.com (Y.L.); 2 Key Laboratory of Instrumentation Science & Dynamic Measurement, North University of China, Taiyuan 030051, China; 3 Chongqing Aerospace Mechanical & Electrical Design Institute, Chongqing 400000, China; 18335162702@163.com

**Keywords:** passive semi-strapdown, isolation rolling platform, high-speed rotation, overload buffer

## Abstract

The isolation rolling platform inside a passive semi-strapdown inertial navigation system can isolate the high-speed rotation of a projectile via bearing to provide a low rotating speed environment for the angular rate sensors inside the platform in order to further improve the accuracy by reducing its measurement range. Aiming at the problem that the internal bearing cannot withstand high overload, an optimal design method for a high overload buffer structure based on point contact spherical cap structure is proposed. Changing the materials of the spherical caps can reduce the deformation of the two spherical caps during impact and reduce the pivoting friction; at the same time, the upper and lower spherical caps are both forced to separate by the spring force after the impact and to eliminate the influence of the pivoting friction torque that is generated by the contact of two spherical caps on the stability of the isolated rolling platform. By virtue of finite element analysis and ground semi-physical simulation experiments, the feasibility of the design is verified. The experiment results show that the design can play an effectively protective role in anti-high overload, and the maximum deformation radius of the optimized point contact spherical cap structure can be reduced by 40.8%; after the upper and lower spherical caps are separated, the isolation rolling platform’ capability of anti-high-speed rotation can be improved by 52% under the rotation speed of the main shaft at 10 r/s. In this way, the stability of the platform is improved, thus proving the value of the design method in engineering applications.

## 1. Introduction

Conventional ammunition plays an irreplaceable role in the modern battlefield [1]. With the development of technology and increasingly complex battlefield environments, the strike mode of traditional artillery has been replaced by precise strike, and various nations have begun to pay attention to studies on guided projectiles and extended range guided ammunition [2,3]. Guided ammunition, with a better precision and range than traditional ammunition, lower costs than conventional ammunition, and higher cost-effectiveness in general, has been widely adopted in modern warfare [4,5]. The precise measurement of navigation information parameters, such as projectile attitude, speed and location, is important for enhancing the precision of guided ammunition. Affected by dimensions and severe environments, it is difficult to apply advanced navigation means, including high-precision inertial components like fiber-optic gyroscopes and image matching, in guided ammunition [6]. Due advantages such as its small size, anti-high overload capacity, strong autonomy, and high reliability, an inertial measurement system based on micro-electro-mechanical system-based inertial components has become an essential navigation component in the guidance process of rotating ammunition with high overload, high impact and short flight time [7,8,9]. However, because of its manufacturing technique, its measurement range and precision are not matched with each other; thus, the attitude of the ammunition when rotating at a high speed cannot be accurately measured by a traditional strapdown inertial navigation system [10]. A gyroscope-free strapdown inertial navigation system is suitable for inertial navigation with a large dynamic range, whereas the angular velocity is obtained from accelerometers’ specific force signals, so the calculated quantity is large and the calculation error quickly accumulates over time, further decreasing accuracy [11,12]. Rotation modulation technology provides stable operating environments via a servo motor. However, because the servo motor has poor resistance to high overload, so the system can only be applied in rocket projectiles with low overload and in high speed rotation [13]. Regarding the limitation of strapdown inertial navigation, the proposal of a passive strapdown inertial navigation system provides a better solution. The micro-inertial measurement unit (MIMU) in the isolated rolling platform of the system maintains a strapdown with the projectile on the pitch and yaw axes. By isolating the projectile’s rolling movement via a mass eccentricity device and bearing, the isolation rolling platform remains stable along the direction of the rolling axis, thus providing an appropriate application environment for MEMS inertial components with a low measurement range and high precision [14,15,16]. The normal operation of the bearing is critical to the rolling of an isolation projectile. However, the anti-overload performance of a bearing is extremely low and easily damaged in the moment of the launch of the ammunition or the in the boosting stage by boost engine because of high overload; thus, a reliable high overload buffer device is of significance to the normal operation of this system.

The anti-high overload protection plan of a traditional strapdown inertial navigation system is to prevent the input of external energy via a vibration isolation buffer material and then perform an encapsulating treatment with internal electronic parts and components. In this way, the system is entirely solidified to be an entirety, so as to prevent the failure of internal parts due to vibration or collision [17,18,19], whereas the core of the passive semi-strapdown inertial navigation system is intended to isolate the high-speed rotation of the projectile body by bearing, Hence, the isolation rolling platform cannot be fixed with an external cylinder. The traditional encapsulating protection method is obviously not suitable for the system. Based on this, an anti-high overload method based on point contact spherical cap structure is proposed here. This method bears the overload that is caused at the moment of shelling via a point contact spherical cap structure that offsets high overload by the deformation of two hemispheres and prevents the direct impact on bearing [20]. This method can avoid damage to the system that is caused by high overload, but the deformation on the top of the two spherical caps provides the point contact to surface contact. Additionally, after the impact, the separation of the two spherical caps cannot be ensured, especially in the engine boost phase of the projectile, and the pivoting friction torque increases, which is not conducive to the stability of the isolation rolling platform.

In view of the fact that the original point contact spherical cap structure cannot effectively separate the upper and lower spherical caps on the extended-range guided shells and reduce the stability of the isolation rolling platform, an optimal design method of the overload buffer structure of a passive semi-strapdown inertial navigation system is proposed in this paper. This method, based on point contact spherical cap structure, changes the materials of two spherical caps and adds a high damping buffer material to reduce the pivoting friction torque in in-bore impact by reducing the deformation of the upper and lower spherical caps. Additionally, this method changes the original point contact method to leave some clearance between two spherical caps, and realizes the separation by virtue of spring force after impact, thereby ensuring that the isolation rolling platform performs compound pendulum movement only under the action of bearing friction torque and gravity recovery torque during the out-of-bore flight phase, so as to enhance the stability of the system. Through a series of theoretical analyses and experimental verifications, the designed method is shown to provide a stable test environment for the MIMU inside and to improve the test accuracy of the system, thus laying the experimental foundation for the application of a passive semi-strapdown inertial navigation system in the guidance of conventional ammunition.

## 2. Compositions and Working Principle of the Passive Semi-Strapdown Inertial Navigation System

### 2.1. Compositions

**Figure 1:**
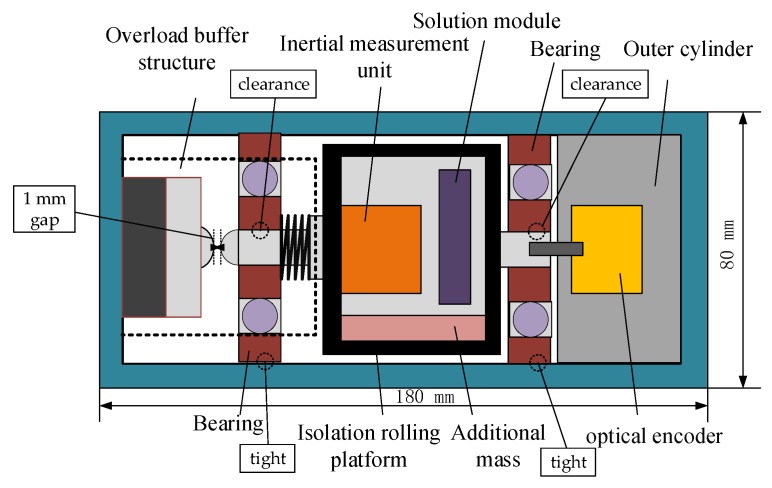
Passive semi-strapdown inertial navigation measurement system.

The semi-strapdown inertial navigation measurement system consists of an outer cylinder that includes a photoelectric encoder, an isolation rolling platform, and a high overload buffer structure at the bottom. The isolation rolling platform carries the MIMU and navigation computer in order to realize center-of-gravity shift by installing the mass block with a small size and large density so as to obtain the gravity restoring torque. Figure 1 is a drawing of the composition of the passive semi-strapdown inertial navigation measurement system.

### 2.2. Working Principle

**Figure 2:**
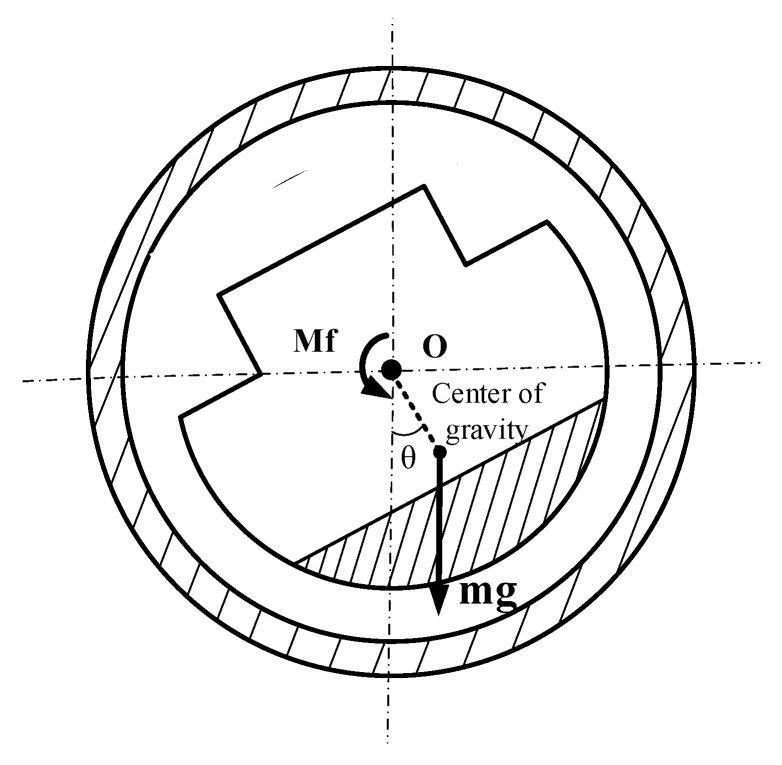
Mechanical model of the semi-strapdown isolation rolling platform.

The core part of passive semi-strapdown inertial navigation measurement unit is the isolation rolling platform, which is designed based on the compound pendulum movement under gravity, the remaining strapdown with the projectile in pitch and yaw bearing, and connection with the projectile in rolling axis via bearing; its mechanical model is shown in Figure 2. The isolation rolling platform retains the stable compound pendulum movement in a balanced position under the friction torque between the gravity restoring torque and the bearing friction torque so as to reach the high-speed rotation of the isolation projectile and provide an appropriate measurement environment for the MIMU with a low measurement range and a high accuracy [21]. The outer cylinder is installed with an optical encoder to measure the relative the angle between the isolation rolling platform and the projectile on the rolling axis [22]. The inertial measurement unite can realize the measurement of movement parameters and the real-time solution of the navigation algorithm on the navigation computer.

## 3. Optimization and Analysis of High Overload Buffer Structure

The movement of the gun-launched missile can be divided into the in-bore launch phase, the intermediate inertial flight phase, the engine boost phase, and the final inertial flight phase. The projectile is mainly subjected to the huge impact that is generated by the powder explosion to obtain kinetic energy at the in-bore launch phase. The intermediate inertia flight phase and the final inertial flight phase are collectively referred to as the inertial flight phase, and, at this stage, the projectile only supports aerodynamics and its own gravitational force. During the engine boost phase, in addition to aerodynamics and its own gravitational force, it is also affected by projectile tail boost engine thrust. The projectile is mainly subjected to axial stress, radial stress, and circumferential stress. Among them, the latter two are very small, no more than one-tenth of the axial stress [23] and far lower than the rated radial load of the deep groove ball bearings. However, the axial stress in the bore launch phase is the largest stress, and the instantaneous acceleration can reach more than 10,000 g. The axial stress is very small in the inertial flight and boost phases, with the maximum not exceeding 10 g. In this regard, it is only required to conduct the axial overload protection of the system, which can be divided into two parts: the in-bore buffer and the out-bore isolation. Among them, the in-bore buffer mainly uses the deformation of the overload buffer device to offset the instantaneous overload during the in-bore launch phase, thereby avoiding the direct impact load on the bearing and damage to the bearing; the out-bore isolation mainly uses external forces to separate the upper and lower spherical caps after collision deformation, thereby eliminating the pivoting friction torque that is caused by the contact between the two spherical caps so that the isolation rolling platform can only perform compound pendulum movement under the bearing friction torque and the gravity restoring torque, which can enhance the stability.

### 3.1. Design of In-Bore Buffer

#### 3.1.1. Principle of In-Bore Buffer

**Figure 3:**
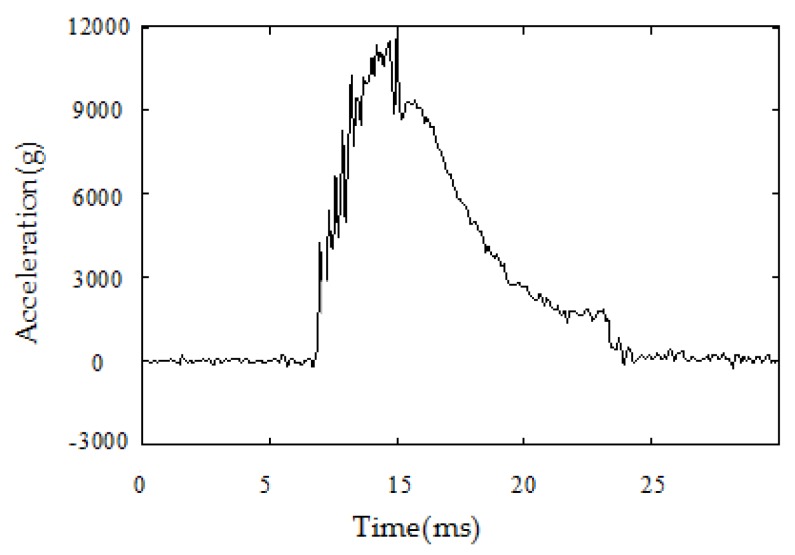
The in-bore acceleration of a 122 mm extended-range guided projectile.

In terms of the in-bore buffer design, it is necessary to first know the overload conditions of the projectile in the bore. Figure 3 is the in-bore acceleration of a certain type of 122 mm extended-range guided projectile. It can be seen that the time for the in-bore overload impacting the projectile is very short, only about 15 ms, but the instantaneous acceleration can reach as high as 12,000 g. The mass of the isolation rolling platform is about 1 kg, and it can generate an impact force of nearly 120,000 N. The bearing type used in this system is SKF6200, the inner diameter is 10 mm, and the static load rating C_0_ is 2.36 kN. According to the SKF Bearing Selection Manual, small bearings with an inner diameter of less than 12 mm must not withstand an axial load exceeding 0.25 C_0_ [24], which is 590 N. Therefore, if the impact force at the moment of shelling directly acts on the bearing, it inevitably leads to irrecoverable damage to the bearing, and it must have an overload buffer structure to bear the impact force to ensure the normal operation of the bearing.

**Figure 4:**
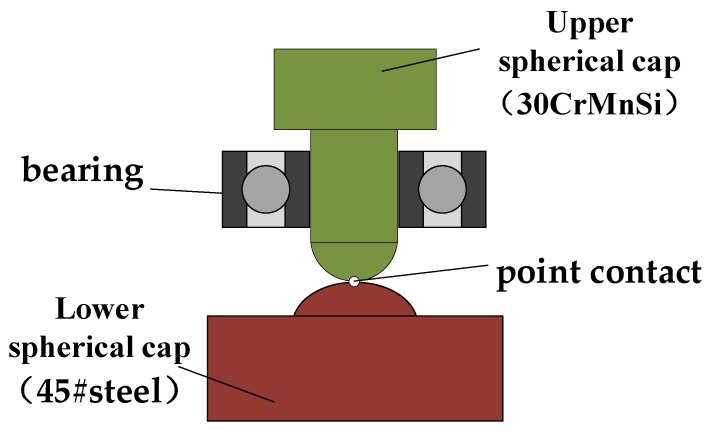
The point contact spherical cap structure.

While bearing the high overload, it is also necessary to ensure that there is not too much friction between the isolation rolling platform and the overload buffer structure in order to avoid generating a pivoting friction torque greater than the gravity restoring torque, which would cause the isolation rolling platform to rotate with the projectile and exceed the angular velocity range of the inertial measurement device. The overload buffer structure in [19] adopted the point contact spherical cap structure shown in Figure 4. The mechanism makes use of the deformation of the upper and lower spherical caps to offset the instantaneous overload. Though this method can effectively resist the impact of high overload on the bearing, there are certain drawbacks, such as the fact that with the increasing amount of deformation, the contact area between the spherical caps and the pivoting friction torque also increase, and this method cannot effectively separate the upper and lower spherical caps during the projectile out-bore flight phase, especially during the engine boost phase, thus reducing the stability of the isolation rolling platform.

**Figure 5:**
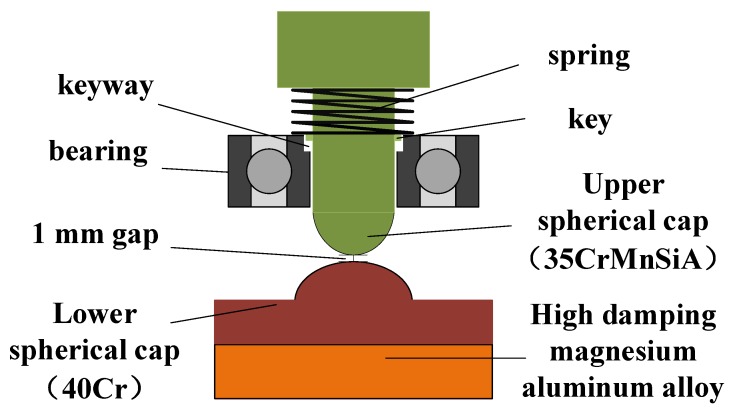
The optimized buffer device.

Aiming at the above problems, the in-bore overload buffer structure was optimized as shown in Figure 5. The isolation rolling platform still withstands the instantaneous high overload via two spherical caps, but the difference is that the original point contact spherical cap structure was changed to non-contact spherical cap structure, leaving a 1 mm gap between the apexes of the two spherical caps and additionally installing a compression spring between the isolation rolling platform shaft and the bearing. When the projectile is in a standstill state or its overload is small, the bearing and the spring provide the supporting force for the isolation rolling platform. At the moment of the shelling, the spring compresses, the two spherical caps come into contact, and the impact load acts on the two spherical caps, thereby preventing the impact on the bearing. In order to prevent excessive friction between the isolation rolling platform shaft and the bearing, and damage to the bearing during the impact, a key connection method was applied between the shaft and the bearing inner ring, the shaft and the inner ring, the key and the keyway must be connected with a certain clearance, and the clearance should not be greater than 0.05 mm, so as to reduce the friction and ensure a high coaxiality of the upper and lower spherical caps. In this way, not only can the isolation rolling platform rotate synchronously with the inner ring of the bearing, but there is also a certain amount of movement allowance in the axial direction that helps to avoid the direct impact of the isolation rolling platform on the bearing.

For the design of the latter hemisphere, it was necessary to reduce the thickness of the column part based on the original design and replace it with high-damping magnesium-based alloy material. The upper and lower spherical caps are made of high-strength, high-elasticity modulus metal materials. Under the instantaneous load at launch, the magnesium-based alloy columns at the bottom of lower spherical cap undergo elastic deformation, absorbing a part of the stress [25] and reducing the deformation of the upper and lower spherical caps, as well as the pivoting friction torque of the upper and lower spherical caps.

#### 3.1.2. The Selection of Materials

According to the requirements, high-strength metal materials should be selected for the upper and lower spherical caps. In order to prevent the cracking of the upper and lower spherical caps instead of deforming under high overload impact, different materials should be chosen for the upper and lower spherical caps to provide better hardness for the upper spherical cap and a better toughness. 35CrMnSiA (Shanghai Meng Teng Metal Materials Co., Ltd., Shanghai, China) is low-alloy high-strength steel, with high strength and sufficient toughness after quenching and tempering, whose yield strength σ_s_ is greater than 1275 MPa [26], but the yield strength of 30CrMnSi is only 835 MPa [27], so 35CrMnSiA is more suitable for the processing of the upper spherical cap. For the lower spherical cap, 45# steel was selected in the traditional point contact spherical cap structure because 45# steel is a high-quality carbon structural steel with relatively low surface hardness and yield strength σ_s_ of only 355 MPa whose deformation at the moment of shelling is relatively large. In this regard, 40Cr can be used instead of 45# steel. 40Cr is an alloy structural steel. Under normal conditions, the mechanical properties of 40Cr are not much different from 45# steel, but after specific heat treatment, its mechanical properties such as strength, hardness, and impact toughness are significantly higher than 45# steel, and its yield strength can reach 785 MPa [28], which is much lower than the yield strength of upper spherical cap, thus ensuring that deformation occurs mainly in the lower spherical cap and is lesser than that of 45# steel. The aluminum–magnesium alloy AZ91D (Shanghai Meng Teng Metal Materials Co., Ltd., Shanghai, China) [29], with good vibration damping performance, is most widely used as the buffer material. Though its damping performance is not as good as that of pure magnesium or rubber materials, its strength is high and it will not cause excessive deformation during impact.

#### 3.1.3. Mechanical Simulation

**Figure 6:**
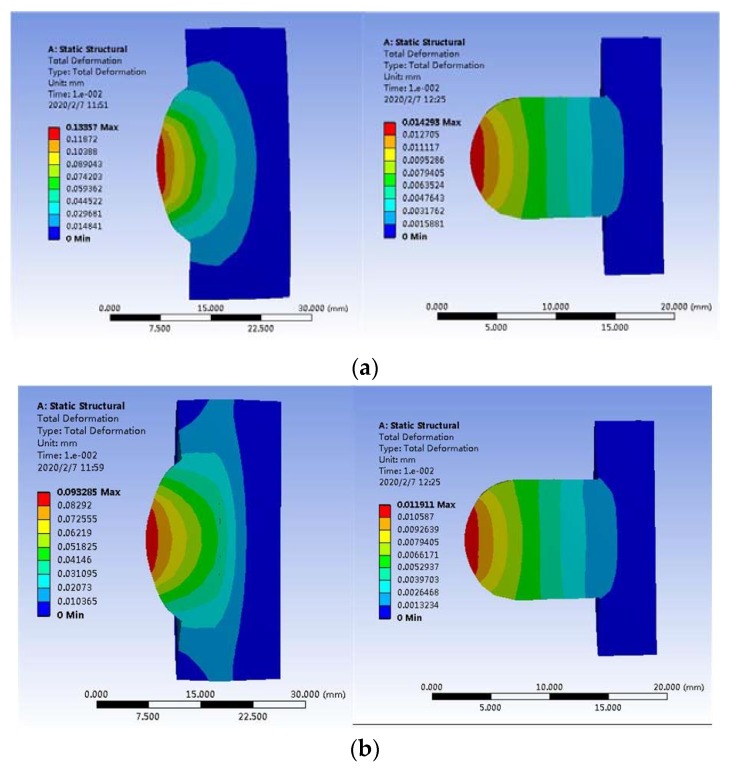
Deformation of upper and the lower caps: (**a**) before optimization and (**b**) after optimization.

In order to simulate the deformation state of the upper and lower spherical caps after the moment of launch, a mechanical simulation was performed. The finite element method was used to establish the simulation model of the point contact spherical cap structure before and after optimization, as well as reasonable free mesh generation. According to the actual projectile environment, at the moment of launch, the projectile must withstand an overload of 12,000 g, because the lower spherical cap is connected to the projectile via the outer cylinder in a fixed way. According to Newton’s second law F = ma, the overload can be made equivalent to the isolation rolling platform by applying the same amount of load force to the lower spherical cap through the upper spherical cap [20]. If the mass of the isolation rolling platform is 1 kg, the equivalent load force is 117,600 N. Figure 6 shows the cloud distribution of the amount of deformation in the upper and lower spherical caps.

From the simulation results, it can be seen that the total deformation of upper and the lower caps after optimization was only 0.10 mm due to the increase in material hardness and yield strength and the buffer effect of the rear aluminum–magnesium alloy, which was 28% less than that before the optimization. In the case of spherical contact, stress concentration occurred at the contact point. Due to the small contact area, the stress generated at the contact point was greater than the yield limit of the materials, so the deformation occurring at the top of the spherical cap was mainly plastic deformation.

### 3.2. The Design of Out-Bore Isolation

#### Design Principle

The design of out-bore isolation was different from that of the in-bore buffer. The purpose was not to resist high overload; rather, it was to stop the upper and lower spherical caps from contacting after impact by spring force, to further eliminate the pivoting friction torque of the two spherical caps to the isolation rolling platform, and to enhance the stability of the projectile. While the spring provides supporting force, it must also ensure that its spring force does not exceed the axial loads that the bearing can withstand to avoid the damage to the bearing. To determine the spring stiffness, it was essential to first analyze the contact state of the two spherical caps during the out-bore flight phase.

**Figure 7:**
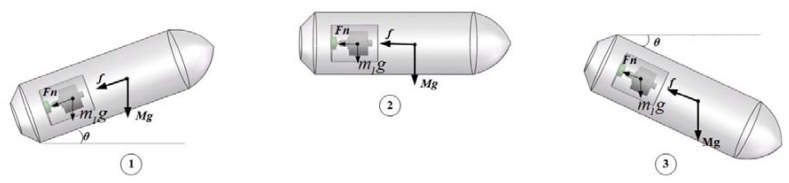
The analysis on affecting force in inertial free flight phase.

During the inertial free flight phase, the projectile is mainly affected by aerodynamics and gravity, of which only the air resistance and the component of projectile gravity force in the axial direction. As shown in Figure 7, f is the axial air resistance of the projectile, Mg is the gravity force acting on the projectile, Fn is the support force of the projectile on the isolation rolling platform in the axial direction, $m_{1}g$ is the gravity force acting on the isolation rolling platform, and *θ* is the projectile pitch angle.

In phase ①, when the projectile pitch angle is positive, the projectile decelerates in the direction of its own rolling axis. At this time, the acceleration is provided by the component at rolling axis of gravity force acting on the projectile and air resistance, and the two forces are in the same direction. In phase ②, the pitch angle is 0, and the projectile only performs deceleration movement under the impact of air resistance. In phase ③, the projectile pitch angle is negative, the projectile performs acceleration motion, and the acceleration is provided by the component of gravity in the direction of the projectile axis and air resistance. Hence, during the inertial free flight phase, Equations (1) and (2) can be used:

(1) Mgsinθ+f=Ma

(2) $$f=\frac{1}{2}C_{x0}\rho V^{2}S\cos\delta$$

In Equation (2), $C_{x0}$ is the axial aerodynamic coefficient, ρ is the air density, V is the velocity of the target relative to the air, S is the maximum cross-sectional area of the projectile, and δ is the projectile angle of attack [30].

For the isolation rolling platform, suppose the front-end bearing provides support for it and it is positive along the tail direction, as shown in Figure 7; as such, Equation (3) can be obtained:

(3) mgsinθ+Fn=ma

From Equations (1) and (3), Equation (4) can be obtained:

(4) $$Fn=\frac{m}{M}f$$

**Figure 8:**
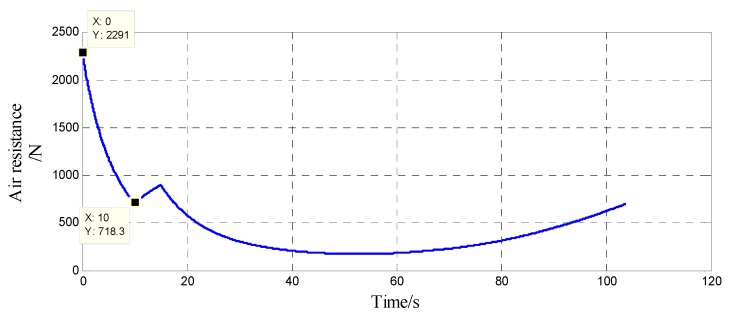
The air resistance of the projectile in out-bore flight.

**Figure 9:**
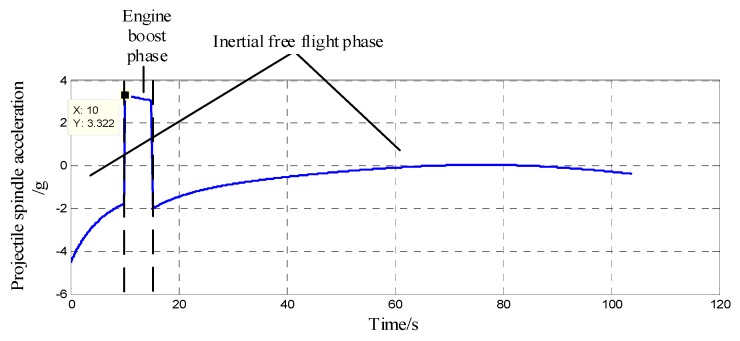
The acceleration of the projectile in out-bore flight.

The Simulink tool in Matlab was used to perform six-degree-of-freedom modeling on a certain model 122 mm extended-range guided projectile. Through simulation, the axial air resistance f and acceleration a of the projectile during the out-bore flight stage were found and are shown in Figure 8 and Figure 9. It can be seen that during the inertia free flight phase, the maximum axial air resistance to the projectile was 2290 N. The mass of the projectile was 60 kg, and the mass of the isolation rolling platform was 1 kg. Additionally, during the inertia free flight phase, the maximum supporting force of the isolation rolling platform was $Fn_{\max}=$ 38.17 N. Since f>0, Fn was always positive, and the supporting force was provided by the front-end bearing; at this time, the upper and lower spherical caps were in a separated state.

**Figure 10:**
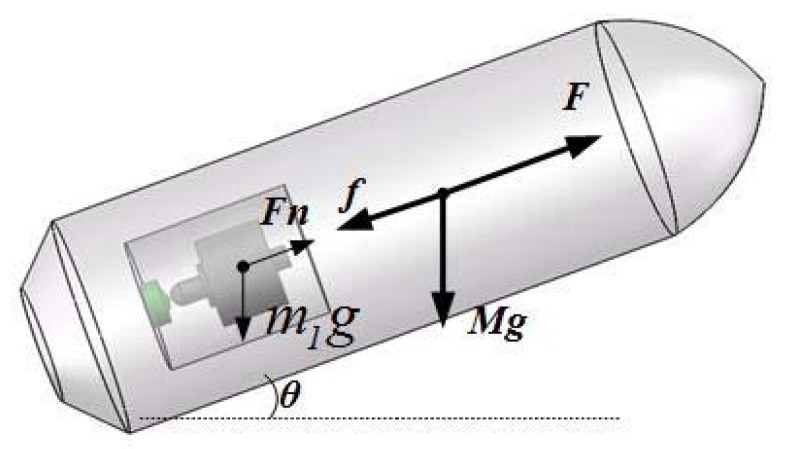
Force analysis of inertial free flight phase.

During the engine boost phase, the projectile is in the acceleration stage under air resistance, the axial components of gravity acting on projectile, and thrust [31], as shown in Figure 10, and Equation (5) can obtained.

(5) F−f−Mgsinθ=Ma

At this time, the supporting force is provided by the lower spherical cap to the isolation rolling platform, and Equation (6) can be obtained:

(6) $$Fn-m_{1}g\sin\theta =m_{1}a$$

By combining Equations (5) and (6), Equation (7) can be obtained:

(7) $$Fn=\frac{m_{1}}{M}(F-f)$$

The thrust F=3000 N. The supporting force of the lower spherical cap on the isolation rolling platform can be obtained from Equation (7), which, in the engine boost phase, is inversely proportional to the air resistance; that is, the support force provided by lower spherical cap when the air resistance is 723 N is maximum, $Fn_{\max}\approx 38.0$ N. Therefore, as long as the spring force that is generated by the spring is greater than 38.0 N, it can replace the lower spherical cap to provide support for the isolation rolling platform and ensure the separation of the upper and lower spherical caps after impact, thereby eliminating the pivoting friction torque during the engine boost phase. At the same time, it is necessary to ensure that the spring force that is generated by the spring in the in-bore buffer stage cannot be greater than the rated axial load of the bearing. Because of the limited space, a compression spring with a spring stiffness of 50 N/mm and a length of 10 mm was selected. From the ANSYS simulation in Section 3.1.3, it can be seen that the maximum amount of deformation of the overload buffer structure at the moment of shelling was 0.10 mm, and the total spring deformation Δx1 was 1.1 mm. According to Hooke’s law, Equation (8) can be obtained:

(8) F=k·Δx

According to Equation (8), the maximum elastic force that can be generated by the spring during the in-bore launch phase $F_{\max}$ = 55 N < 590 N, which is within the rated axial load of the bearing, so it cannot cause damage to the bearing. The maximum deformation of the spring during the out-bore inertial flight phase $\Delta x=\frac{Fn_{\max}}{k}\approx$ 0.76 mm < Δx1, so the upper and lower spherical caps will not contact each other.

## 4. Test Verification and Discussion

In order to verify the actual performance of this high overload buffer structure, the high overload and high-speed rotation environment of the projectile during the launch was simulated, and a ground semi-physical simulation experiment was performed.

### 4.1. Impact Test

**Figure 11:**
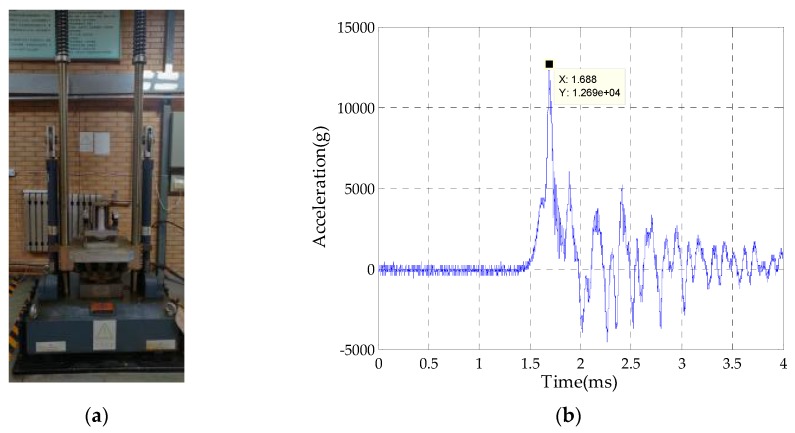
The scene diagram of impact test. (**a**) System installation photo and (**b**) impact table feedback waveform.

**Figure 12:**
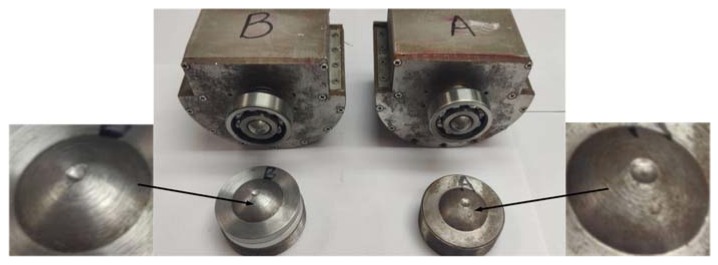
The condition of each component after the impact.

First of all, in order to verify the anti-high overload performance of the optimized passive semi-strapdown inertial navigation system, ground semi-physical tests were separately performed on the two systems, and the high overload environment in the bore of the projectile at the moment of the projectile launch was simulated by a hydraulic impact table. An original anti-high overload structure was used for system A, and an optimized overload buffer structure was used for system B. During the experiment, the system was powered on for 10 s to ensure that the internal circuits and sensors were in normal working state, and then the magnitude of the load table of the hydraulic impact table was adjusted so that it could generate an impact overload of about 12,000 g, and the impact tests were performed on systems A and B in turn. Figure 11 is a scene diagram of the impact test, and the state of each component after the impact is shown in Figure 12.

After the impact, the bearings of both systems rotated smoothly without damage. The deformation of the upper spherical caps of both systems was very small and could be ignored, while the deformation of the two lower spherical caps was quite different. The deformation of the lower spherical cap of system B was significantly smaller than that of system A. After the measurement, the deformation of system A was about 0.15 mm, and its maximum deformation radius was 1.79 mm. However, the deformation of system B was only 0.09 mm, and its maximum deformation radius was 1.06 mm, which was basically consistent with the simulated structure. Since the maximum deformation radius of the lower spherical cap of system B was 40.8% smaller than that of system A, it could greatly reduce the pivoting friction that is generated by the contact between the two spherical caps during the in-bore launch phase.

### 4.2. Accelerated, High-Speed Rotation Test

**Figure 13:**
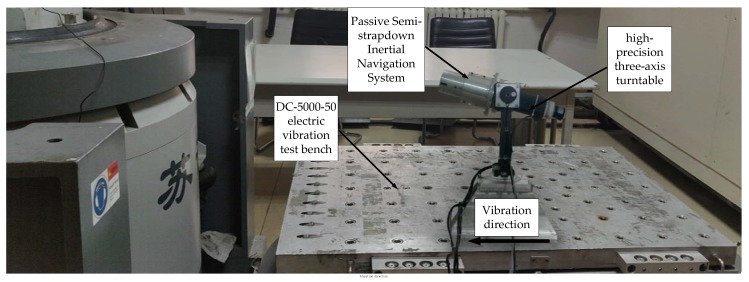
The projectile’s high-rotation and acceleration environment simulation experiment.

**Figure 14:**
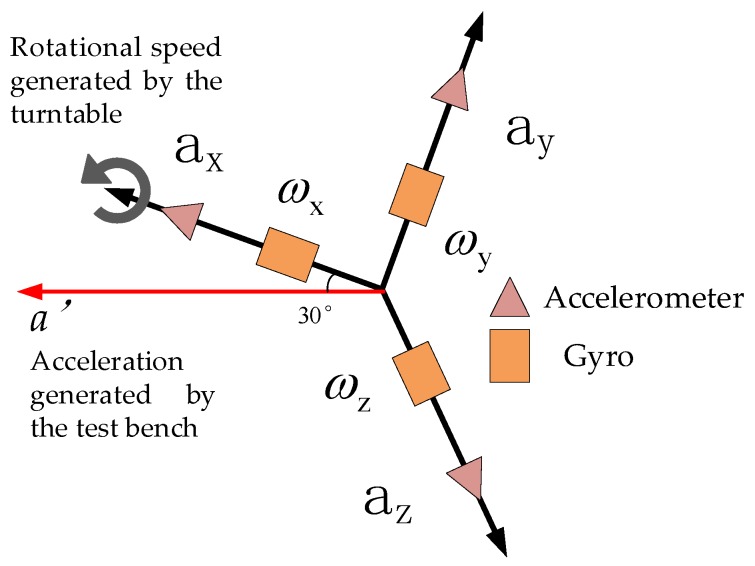
Schematic diagram of the orientation of the sensors inside the isolated rolling platform.

Secondly, to verify the stability of the isolation rolling platform after impact, that is during the out-bore flight phase, this test used a high-precision three-axis turntable to provide the system with axial angular velocity to simulate the projectile’s high-rotation environment during flight. According to the acceleration simulation curve of the projectile during the out-bore flight in Figure 9, the acceleration of the projectile during the boost phase could reach 3.32 g, whereas the acceleration of the ground vehicle was much lower than 1 g. Therefore, a DC-5000-50 electric vibration test bench was used to provide the system with linear acceleration to simulate the projectile’s acceleration during the engine boost phase. The two passive semi-strapdown inertial navigation systems before and after optimization were installed on the high-precision three-axis turntable spindle, the turntable pitch angle was set to 30°, and then the high-precision three-axis turntable was fixed to the vibration bench surface, as shown in Figure 13 and Figure 14 is a schematic diagram of the orientation of the sensors inside the isolated rolling platform; among them, the accelerometers and gyroscopes on three axes were orthogonal to each other.

**Figure 15:**
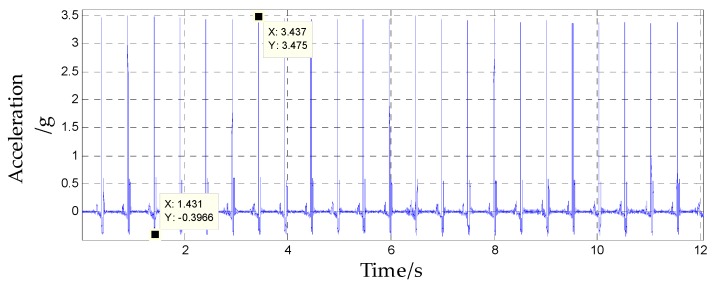
Acceleration of the system spindle direction.

**Figure 16:**
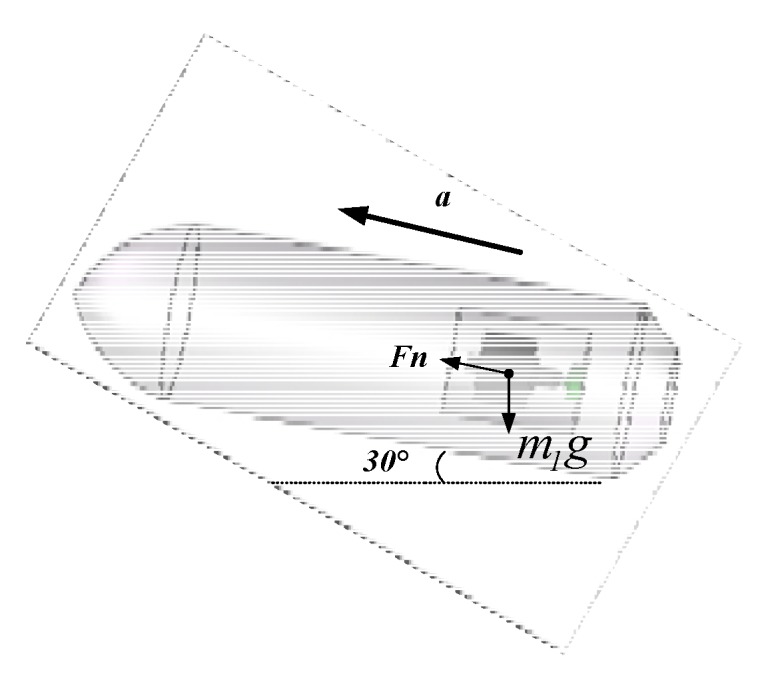
Force analysis of the isolation rolling platform.

After the system was powered on, it was set at rest for 10 s to ensure that the internal sensors worked stably. Then, the vibration bench was started, the turntable spindle was set to rotate counterclockwise at an angular rate of 3600°/s, and the vibration bench was set to perform a half-sine wave vibration in the horizontal direction at a frequency of 2 Hz and a vibration amplitude of 4 g. The acceleration component of the vibration bench in the system spindle direction is shown in Figure 15, and the direction was upward along the system axis. The force analysis of the internal isolation rolling platform is shown in Figure 16.

By assuming that the support force *Fn* of the isolation rolling platform is upward along the system axis, Equation (9) can be obtained:

(9) $$Fn-m_{1}g\sin30=m_{1}a$$

In Equation (9), *g* = 9.8 m/s^2^ and *m*_1_ = 1 kg is the mass of the isolated rolling platform. Then:

(10) $$Fn=m_{1}(g\sin30+a)$$

**Figure 17:**
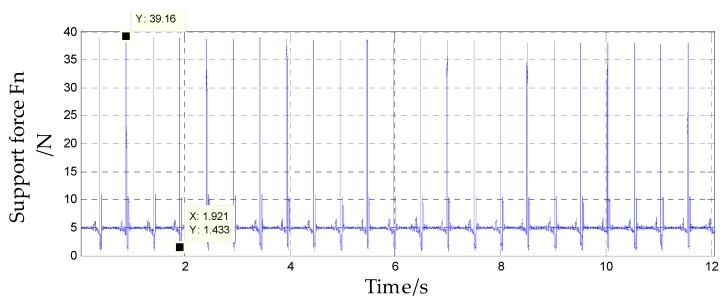
Support force of the isolated rolling platform.

The support force of the isolated rolling platform can be obtained from Equation (10), as shown in Figure 17. Since *Fn* > 0, the assumption was true. In system A, the isolated rolling platform was supported by the lower spherical cap, but in system B, the spring was used to support the isolated rolling platform.

**Figure 18:**
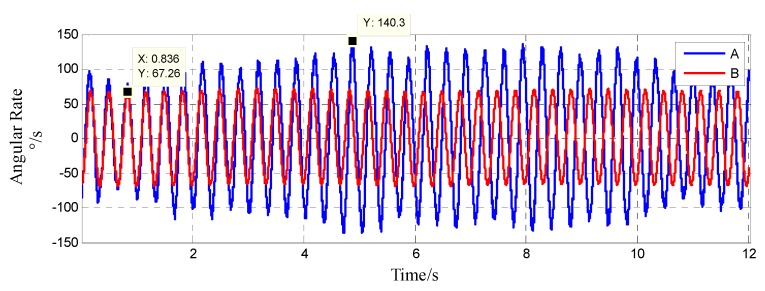
The comparison of the angular rate of axial gyroscope $\omega_{x}$ inside the isolated rolling platform.

Figure 18 shows the feedback waveform of the spindle angular rate sensors of systems A and B. According to the angular rate output curve, under the system spindle’s rotation at an angular rate of 3600 °/s and an acceleration range of −0.39 to 3.47 g, the internal isolation rolling platforms of systems A and B performed compound pendulum movement. The difference was that the angular rate of the internal isolation rolling platform of system A was reduced to a maximum of 140.3 °/s and fluctuated continuously, which indicated that system A was affected by torque in addition to the gravity restoring torque and the bearing friction torque—the pivoting friction torque that was generated by the contact of the upper and lower spherical caps, which caused the amplitude of the compound pendulum to continuously change and the angular rate to change accordingly. However, the angular rate of the internal isolation rolling platform of system B reached a maximum value of 67.26 °/s, and the angular rate change curve was stable, which indicates that the isolation rolling platform only performed the compound pendulum movement under the gravity restoring torque and the bearing rolling friction torque.

## 5. Conclusions

In this paper, the optimization design of the high overload buffer structure of a passive semi-strapdown inertial navigation system was introduced, and the optimized overload buffer structure is analyzed by a mechanical simulation and a ground semi-physical simulation experiment to analyze and verify the performance of the overload buffer structure. By changing the materials of the point contact spherical cap structure, the maximum deformation radius of the two spherical caps during impact was reduced, thereby reducing the pivoting friction that was generated by the contact between the two spherical caps during impact. Taking a certain type of 122 mm extended-range guided projectile as an example, the force of the isolated rolling platform during each flight phase of the projectile was analyzed. For the first time, the external force of the spring was used to force the separation of the upper and lower spherical caps after the impact. This ensured that after impact, the isolation rolling platform only performed compound pendulum movement under the effect of the bearing friction torque and the gravity recovery torque, and it also helped to avoid the pivoting friction torque that was generated by the contact between the two spherical caps to affect the stability of the isolation rolling platform, thereby reducing the capability of anti-high-speed rotation of the isolation rolling platform.

Experiment results showed that the optimized overload buffer structure could withstand 12,690 g overload impact, effectively protect the bearing and the circuits and sensors inside the isolation rolling platform, and reduce the maximum deformation radius of the upper and lower spherical caps reduce by 40.8% more than that before optimization, Additionally, after the impact, in the acceleration range of −0.39 to 3.47 g, the external force that was generated by the compression spring could realize the separation of the upper and lower spherical caps, thus providing a stable and reliable working environment for the internal sensors, and the isolation rolling platform’s capability of anti-high-speed rotation was improved by 52%. This design provides an effective method for the passive semi-strapdown inertial navigation system to better adapt to a complex on-board environment, and it is of important significance in improving the stability and reliability of the isolation rolling platform in extended-range guided projectiles.

## References

[1] Duan X.M., Liu J., Li J. (2014). Design and Test of platform with partial strapdown inertial navigation system for guided projectile. Missiles Space Veh..

[2] Jamshaid A., Fang J.C. (2009). Realization of an autonomous integrated suite of strapdown astro-inertial navigation systems using unscented particle filtering. Comput. Math. Appl..

[3] Du J., Guo Y., Lin Y., Zheng X., Jin Z. A real-time temperature compensation algorithm for a force-rebalanced MEMS capacitive accelerometer based on resonant frequency. Proceedings of the 2017 IEEE 12th International Conference on Nano/Micro Engineered and Molecular Systems (NEMS).

[4] Hou M., Yan K., Wang W. (2017). Status and development trend of long-range guided projectile technology. Aerodyn. Missile J..

[5] Mu Y., Cheng Z.X., Wang J. (2008). Status and Development Trend of Guided Projectile Technology. Aerodyn. Missile J..

[6] Cao F., Yang X.G., Miao D., Zhang Y.P. (2005). Study on Reference Image Selection Roles for Scene Matching Guidance. Appl. Res. Comput..

[7] Wang W., He S. (2009). Development of MEMS inertial instrument technology. Missile Space Veh..

[8] Guo D. (2019). Weapon-target assignment for multi-to-multi interception with grouping constraint. IEEE Access.

[9] Bao Y.Q., Chen G.G., Wu K., Wang X.R. (2008). Research on Attitude Determination Using Magnetometers and MEMS Inertial Sensors. Acta Armamentarii.

[10] Zhang X., Li J., Hou L.-P., Zhu J.-D., Qin L. (2015). Analysis and Compensation of Installation Axial Angle Errors of Semi-strapdown IMS. Acta Armamentarii.

[11] Chen J.-H., Lee S.-C., DeBra D.B. (1994). Gyroscope Free Strapdown Inertial Measurement Unit by Six Linear Accelerometers. J. Guid. Control Dyn..

[12] Dehghani M., Kharrati H., Seyedarabi H., Baradarannia M. (2019). The Correcting Approach of Gyroscope-Free Inertial Navigation Based on the Applicable Topological Map. J. Comput. Inf. Sci. Eng..

[13] Zhang J., Li J., Che X., Zhang X., Hu C., Feng K., Xu T. (2019). The Optimal Design of Modulation Angular Rate for MEMS-Based Rotary Semi-SINS. Micromachines.

[14] Duan X.M., Li J., Liu J. (2014). Research on the Dynamic Model of a Partial Strapdown Platform and the Impact Analysis of Pitching Angle and the Stability of Platform. Acta Armamentarii.

[15] Li J., Zhao Y., Liu J., Chen W. (2013). Research on Semi-strapdown MEMS Inertial Measurement Device for Flight Attitude Measurement of High-speed Rotating Ammunition. Acta Armamentarii.

[16] Li J., Jing Z.Y., Zhang X., Zhang J., Li J., Gao S., Zheng T. (2018). Optimization Design Method of a New Stabilized Platform Based on Missile-borne Semi-Strap-down Inertial Navigation System. Sensors.

[17] Zhang J., Jia H.G., Hao X.Y., Zhou L. (2012). Optimal Design of Buffered Isolation Structure of High Overload Data Storage. Explos. Shock Waves.

[18] Hu C.J. (2015). Design of Micro Inertial Measurement System for Miniature Projectile-Based Equipments against High Overload. Master’s Thesis.

[19] Zhao X.Z. (2008). Anti-High-Overload Design and Application for Electronic Recording System. Master’s Thesis.

[20] Wei X.K., Li J., Zheng T., Zhang X., Feng K.Q., Qian H.N. (2019). Design of anti-overload structure for passive semi-strapdown stable platform. Explos. Shock Waves.

[21] Wei X.K., Li J., Zhang D., Feng K., Zhang J., Li J., Lu Z. (2019). Optimization of a New High Rotary Missile-Borne Stabilization Platform. Sensors.

[22] Xu T.J., Li J., Du S.Y., Zheng T., Wei X., Zhang J. (2018). A New Measurement System for the Rolling Angle of the High-Rotating Projectile. Chin. J. Sens. Actuators.

[23] Wei W., Wang N.F. (2004). Numerical Simulation of Structural Integrity for Solid Propellants under Axial High Overloads. Chin. J. Explos. Propellants.

[24] Originality Document. https://max.book118.com/html/2018/0828/5013044333001311.shtm.

[25] Li J., Ning Q.L., Zhu J.S., Liu C. (2013). Effect of load-relieving material on projectile-based equipments against high overload. Ordnance Mater. Sci. Eng..

[26] Gong L., Zhao W., Ren F., He N., Li L., Xu Q., Khan A.M. (2019). Experimental study on surface integrity in cryogenic milling of 35CrMnSiA high-strength steel. Int. J. Adv. Manuf. Technol..

[27] Nekouei R.K., Akhaghi R., Ravanbkhsh A., Tahmasebi R., Moghaddam A.J., Mahrouei M. (2016). A study of the effect of two-stage tempering on the mechanical properties of steel 30CrMnSi using analysis of response surface in design of experiment. Metal Sci. Heat Treat..

[28] Ge Y.L., Ding Y.C. (2017). The Influence of Heat Treatment Process on the Micro-structure and Mechanical Properties of 40Cr. J. Chengdu Technol. Univ..

[29] Liu X.L., Liu C.M., Chang Y.Z., Liu S.M. (2008). Cesearch Status and Development Progress of High-Damping and High-Strength Magnesium Alloys. Foundry.

[30] Han Z.P. (2014). Exterior Ballistics of Projectiles and Rockets.

[31] Wang Y.H., Li J., Liu W., Zheng T., Du S.Y. (2017). Analysis and Experimental Verification of Stability of the Roll Stabilization Platform on Missile. Sci. Technol. Eng..

